# Supplementary material for: Persistent T cell unresponsiveness associated with chronic visceral leishmaniasis in HIV-coinfected patients
Source: Commun Biol. 2024 May 3;7:524. doi: 10.1038/s42003-024-06225-2 (PMC11068874; doi:10.1038/s42003-024-06225-2)
Supplement: Supplementary file 2 — Supplementary Material [file 42003_2024_6225_MOESM2_ESM.docx]

**Persistent T cell unresponsiveness associated with chronic visceral leishmaniasis in HIV-coinfected patients**

Nicky de Vrij^1,2^, Julia Pollmann^3^, Antonio M. Rezende^4^, Ana V. Ibarra-Meneses^5^, Thao-Thy Pham^1^, Wasihun Hailemichael^6^, Mekibib Kassa^7^, Tadfe Bogale^7^, Roma Melkamu^7^, Arega Yeshanew^7^, Rezika Mohammed^7^, Ermias Diro^7^, Ilse Maes^8^, Malgorzata A. Domagalska^8^, Hanne Landuyt^9^, Florian Vogt^10,11,12^, Saskia van Henten^12^, Kris Laukens^2^, Bart Cuypers^2^, Pieter Meysman^2^, Hailemariam Beyene^13^, Kasaye Sisay^13^, Aderajew Kibret^13^, Dagnew Mersha^13^, Koert Ritmeijer^14^, Johan van Griensven^12^, Wim Adriaensen^1^*

*1Clinical Immunology Unit, Department of Clinical Sciences, Institute of Tropical Medicine, 2000 Antwerp, Belgium*

*2Adrem Data Lab, Department of Computer Science, University of Antwerp, 2020 Antwerp, Belgium*

*3Department of Medical Oncology, University Hospital Heidelberg, National Center for Tumor Diseases (NCT) Heidelberg, 69120 Heidelberg, Germany*

*4Department of Microbiology, Aggeu Magalhães Institute – FIOCRUZ/PE, Recife, Brazil*

*5Département de pathologie et microbiologie, Faculté de médecine vétérinaire, Université de Montréal. Saint-Hyacinthe, QC, Canada.*

*6Department of Immunology and Molecular Biology, Faculty of Biomedical Sciences, University of Gondar, Ethiopia*

*7Leishmaniasis Research and Treatment Centre, University of Gondar, Gondar, Ethiopia*

*8Molecular Parasitology Unit, Department of Biomedical Sciences, Institute of Tropical Medicine, 2000 Antwerp, Belgium*

*9Clinical Trial Unit, Department of Clinical Sciences, Institute of Tropical Medicine, 2000 Antwerp, Belgium*

*10National Centre for Epidemiology and Population Health, The Australian National University, Canberra 2601, Australia*

*11The Kirby Institute, University of New South Wales, Sydney 2052, Australia*

*12Unit of Neglected Tropical Diseases, Department of Clinical Sciences, Institute of Tropical Medicine, 2000 Antwerp, Belgium*

*13Médecins Sans Frontières, Abdurafi, Ethiopia*

*14Médecins Sans Frontières, Amsterdam, The Netherlands*

# Supplementary Figures


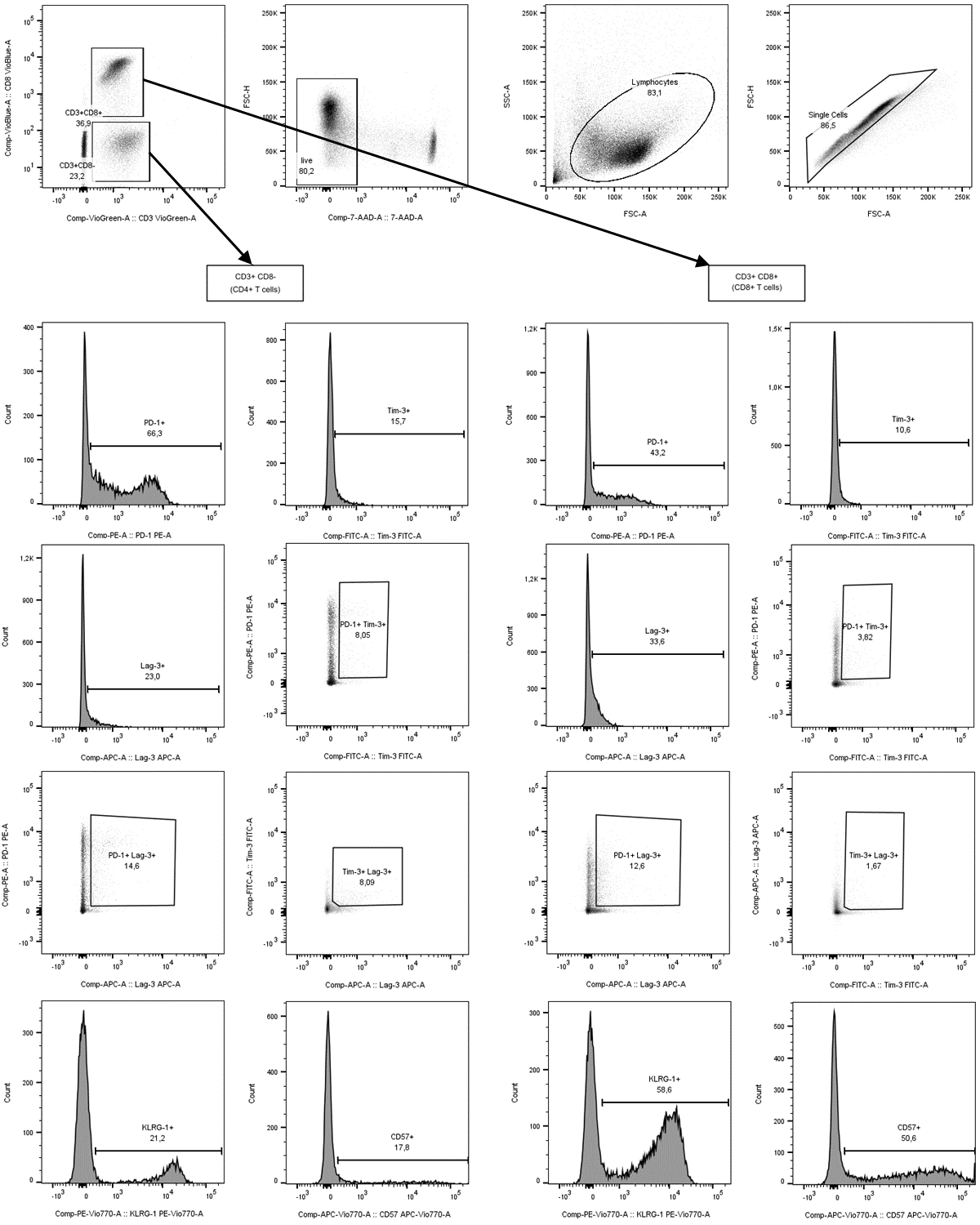


**Supplementary Figure 1 | Gating strategy for the CD8^+^ T cell exhaustion panel.** Patient PBMCs were isolated and stained for flow cytometry. After exclusion of doublets and non-lymphocytes, single lymphocytes were gated for live cells. Next, CD8^+^ and CD8^-^ T cells were gated out by plotting CD3-VioGreen versus CD8-VioBlue. Out of these subsets, those positive for a range of exhaustion and senescence markers (CD57-APC-Vio770, LAG3-APC, KLRG1-PE-Vio770, PD1-PE, TIM3-VioBright-FITC) were gated out. Double positives for any combination of LAG3-APC, PD1-PE, and TIM3-VioBright-FITC were gated out as well.


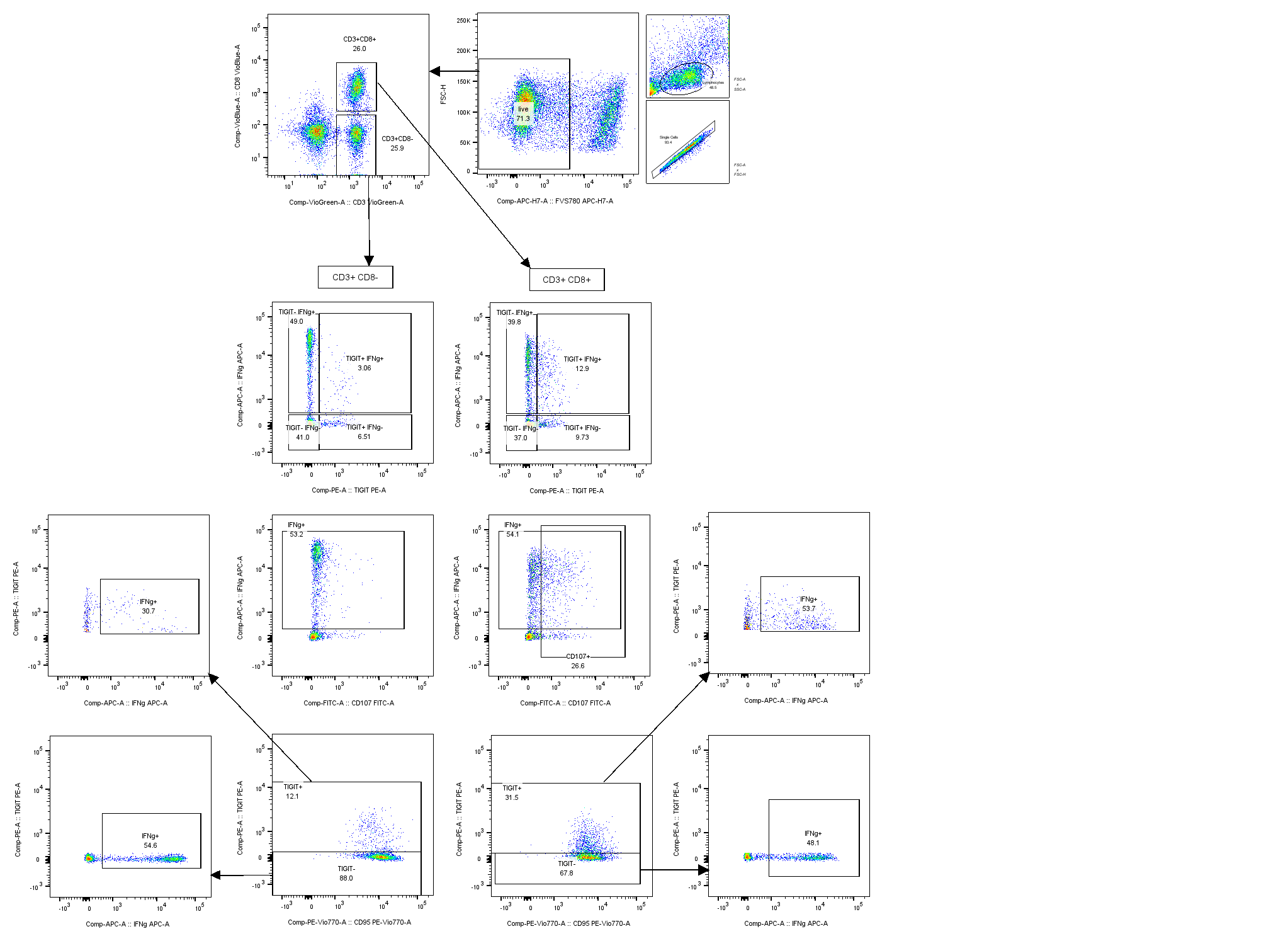


**Supplementary Figure 2 | Gating strategy for the CD8^+^ functionality-targeted panel.** Patient PBMCs were isolated and stained for flow cytometry. After exclusion of doublets and non-lymphocytes, single lymphocytes were gate for live cells. Next, CD8^+^ and CD8^-^ T cells were gated out by plotting CD3-VioGreen versus CD8-VioBlue. Out of these subsets, those positive for TIGIT-PE, IFNy-Vio667, and CD107a-FITC were gated out.

**
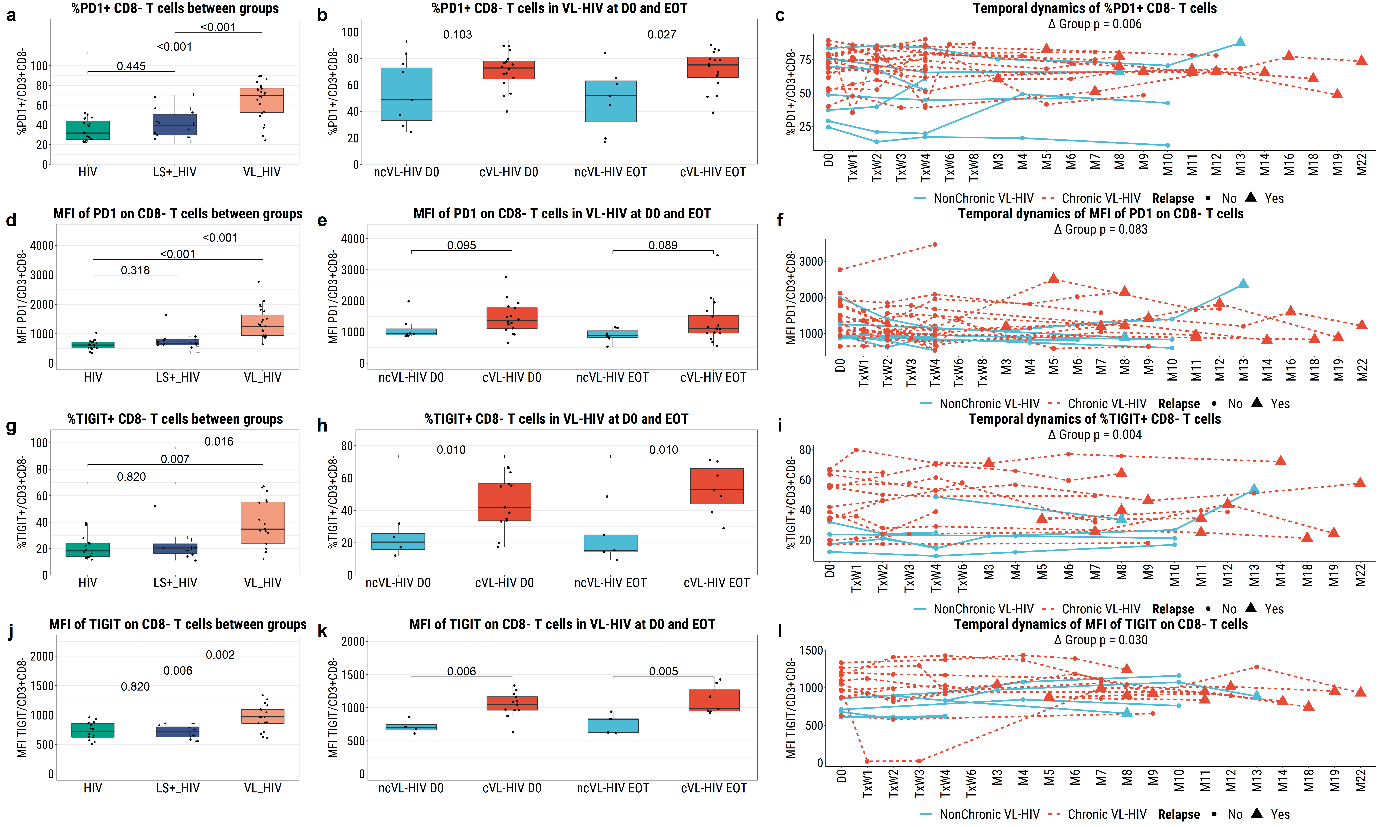
**

**Supplementary Figure 3 | The PD1^+^ and TIGIT^+^ CD8^-^ (CD3^+^CD8^-^) T cell fractions of PBMCs isolated from the different participant groups as measured by flow cytometry.** **(A, D, G, J)** Cross-sectional profiling of the proportion of PD1^+^ CD8^-^ T cells, the MFI of PD1 on CD8^-^ T cells, the proportion of TIGIT^+^ CD8^-^ T cells, and the MFI of TIGIT on CD8^-^ T cells, respectively, between the VL-HIV, the *Leishmania*-seropositive HIV, and the HIV-only groups, using a Benjamini-Hochberg corrected pairwise Mann-Whitney U test to test for statistical differences. In the latter group, the plus sign (+) indicates those *Leishmania*-seropositive individuals with a history of VL. **(B, E, H, K)** Comparison between chronic and non-chronic VL-HIV patients at the active disease development (D0) and End-of-Treatment (EOT) timepoints using a BH-corrected Mann-Whitney U to test for statistical differences **(C, F, I, L)** Longitudinal characterisation of the proportions and MFIs of the same cellular subsets, for non-chronic or chronic VL-HIV patients only, using linear mixed-effects models (see Methods). Cross-sectional analysis total N = 62. Longitudinal analysis N = 24.


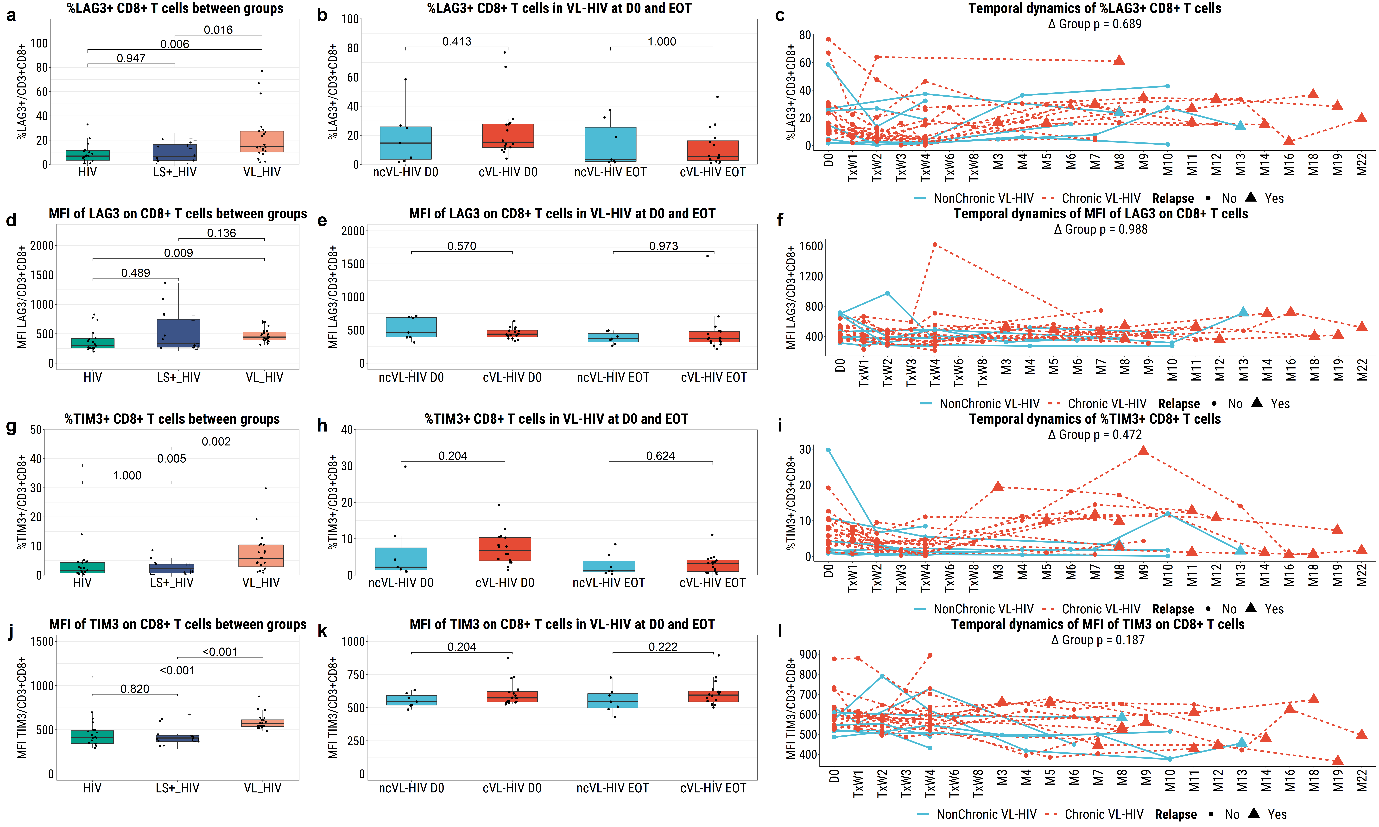


**Supplementary Figure 4 | The LAG3^+^ and TIM3^+^ CD8^+^ (CD3^+^CD8^+^) T cell fractions of PBMCs isolated from the different participant groups as measured by flow cytometry.** **(A, D, G, J)** Cross-sectional profiling of the proportion of LAG3^+^ CD8^+^ T cells, the MFI of LAG3 on CD8^+^ T cells, the proportion of TIM3^+^ CD8^+^ T cells, and the MFI of TIM3 on CD8^+^ T cells, respectively, between the VL-HIV, the *Leishmania*-seropositive HIV, and the HIV-only groups, using a Benjamini-Hochberg corrected pairwise Mann-Whitney U test to test for statistical differences. In the latter group, the plus sign (+) indicates those *Leishmania*-seropositive individuals with a history of VL. **(B, E, H, K)** Comparison between chronic and non-chronic VL-HIV patients at the active disease development (D0) and End-of-Treatment (EOT) timepoints using a BH-corrected Mann-Whitney U to test for statistical differences **(C, F, I, L)** Longitudinal characterisation of the proportions and MFIs of the same cellular subsets, for non-chronic or chronic VL-HIV patients only, using linear mixed-effects models (see Methods). Cross-sectional analysis total N = 62. Longitudinal analysis N = 24.


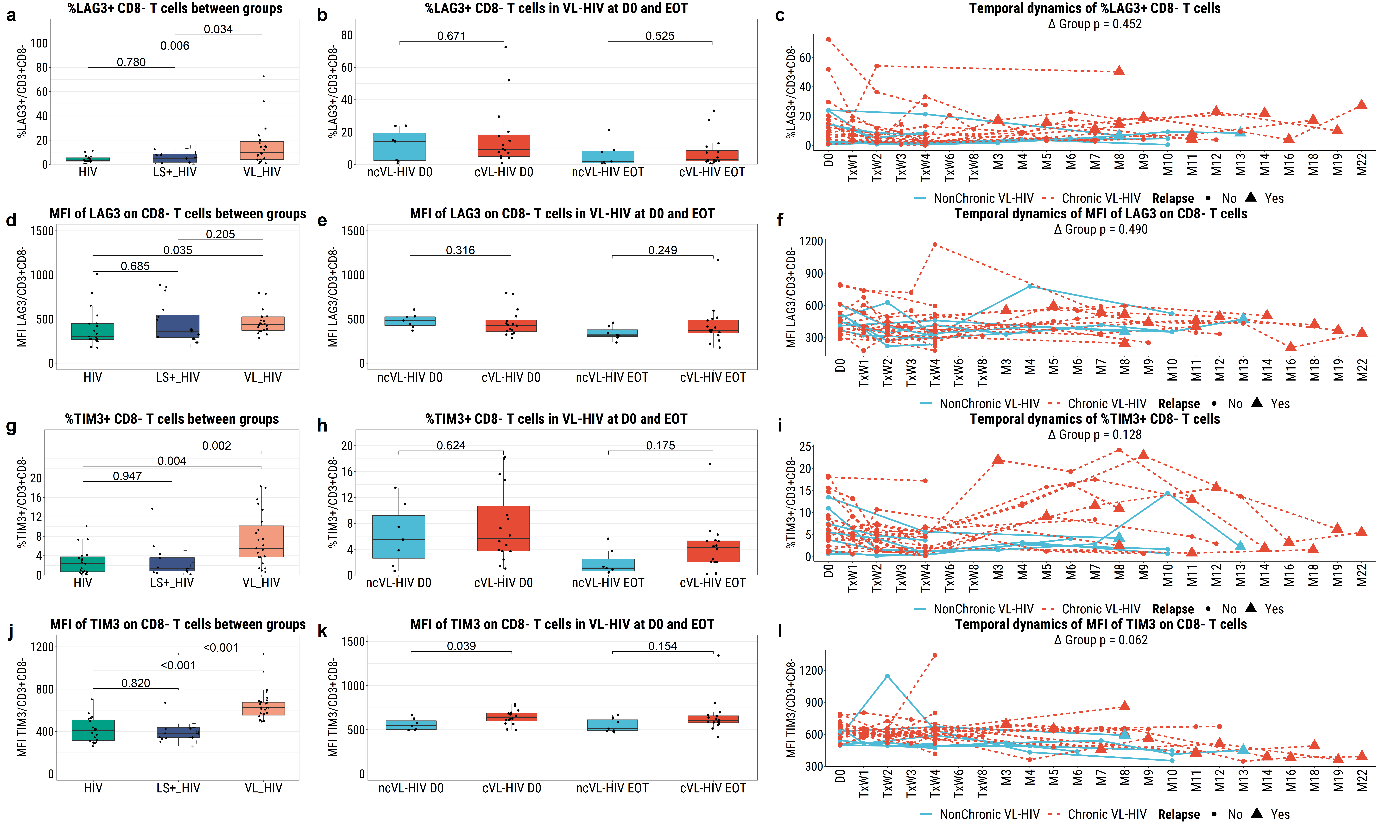


**Supplementary Figure 5 | The LAG3^+^ and TIM3^+^ CD8^+^ (CD3^+^CD8^-^) T cell fractions of PBMCs isolated from the different participant groups as measured by flow cytometry.** **(A, D, G, J)** Cross-sectional profiling of the proportion of LAG3^+^ CD8^-^ T cells, the MFI of LAG3 on CD8^-^ T cells, the proportion of TIM3^+^ CD8^-^ T cells, and the MFI of TIM3 on CD8^-^ T cells, respectively, between the VL-HIV, the *Leishmania*-seropositive HIV, and the HIV-only groups, using a Benjamini-Hochberg corrected pairwise Mann-Whitney U test to test for statistical differences. In the latter group, the plus sign (+) indicates those *Leishmania*-seropositive individuals with a history of VL. **(B, E, H, K)** Comparison between chronic and non-chronic VL-HIV patients at the active disease development (D0) and End-of-Treatment (EOT) timepoints using a BH-corrected Mann-Whitney U to test for statistical differences **(C, F, I, L)** Longitudinal characterisation of the proportions and MFIs of the same cellular subsets, for non-chronic or chronic VL-HIV patients only, using linear mixed-effects models (see Methods). Cross-sectional analysis total N = 62. Longitudinal analysis N = 24.

**
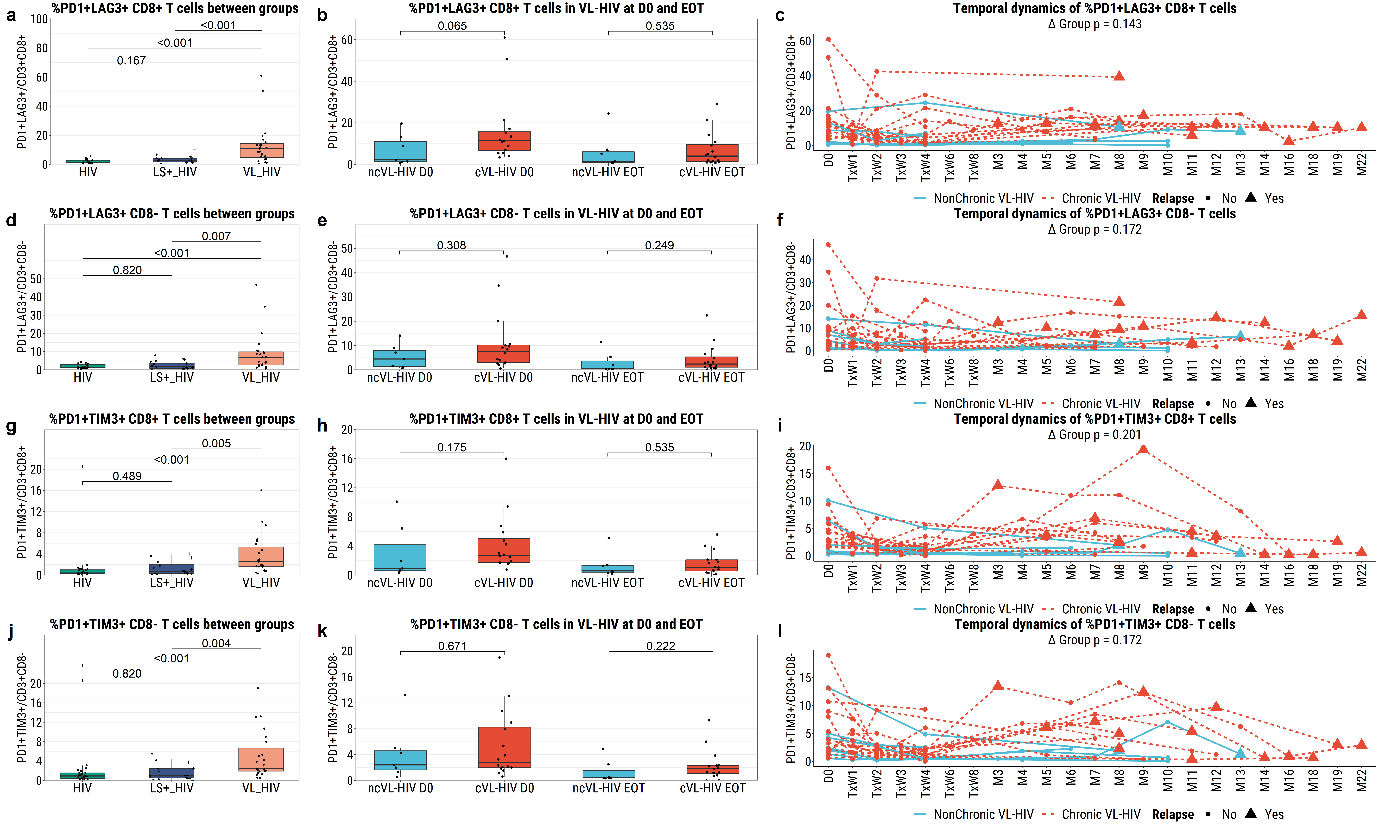
**

**Supplementary Figure 6 | The proportions of cells double-positive for any combination of PD1 -and TIM3 -or LAG3-positive CD8^+^ (CD3^+^CD8^+^) and CD8^-^ (CD3^+^CD8^-^) T cell fractions of PBMCs isolated from the different participant groups as measured by flow cytometry.** **(A, D, G, J)** Cross-sectional profiling of the proportions of PD1^+^LAG3^+^ CD8^+^ T cells, PD1^+^LAG3^+^ CD8^-^ T cells, PD1^+^TIM3^+^ CD8^+^ T cells, and PD1^+^TIM3^+^ CD8^-^ T cells, respectively, between the VL-HIV, the *Leishmania*-seropositive HIV, and the HIV-only groups, using a Benjamini-Hochberg corrected pairwise Mann-Whitney U test to test for statistical differences. In the latter group, the plus sign (+) indicates those *Leishmania*-seropositive individuals with a history of VL. **(B, E, H, K)** Comparison between chronic and non-chronic VL-HIV patients at the active disease development (D0) and End-of-Treatment (EOT) timepoints using a BH-corrected Mann-Whitney U to test for statistical differences **(C, F, I, L)** Longitudinal characterisation of the proportions of the same cellular subsets, for non-chronic or chronic VL-HIV patients only, using linear mixed-effects models (see Methods). Cross-sectional analysis total N = 62. Longitudinal analysis N = 24.


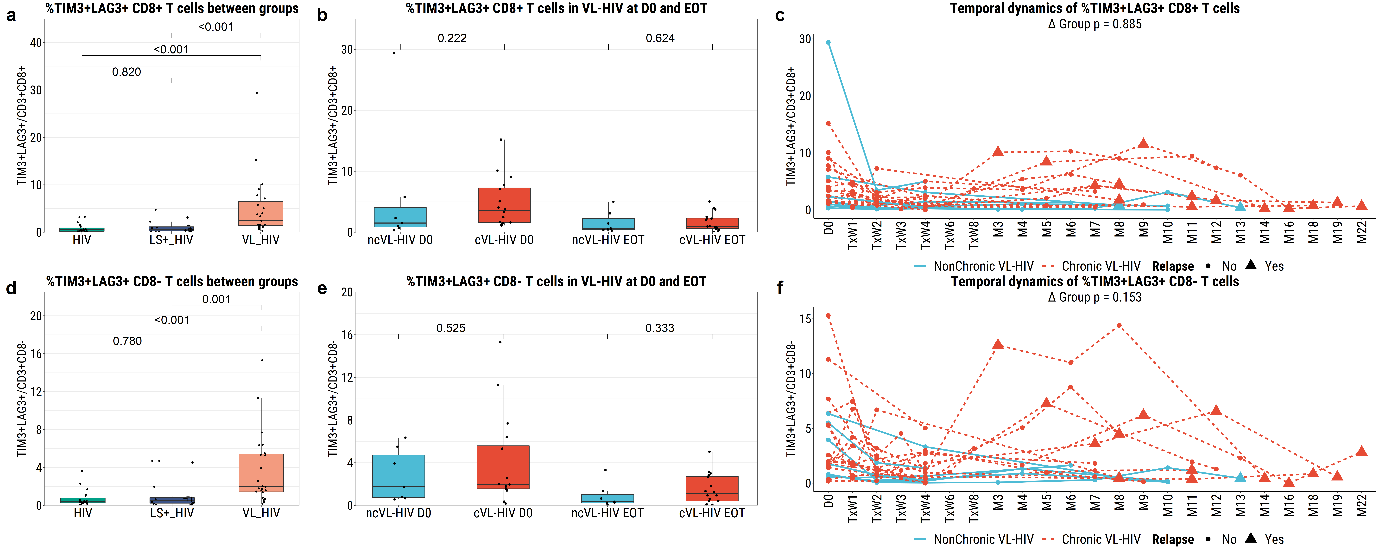


**Supplementary Figure 7 | The proportions of TIM3^+^LAG3^+^ CD8^+^ (CD3^+^CD8^+^) and CD8^-^ (CD3^+^CD8^-^) T cell fractions of PBMCs isolated from the different participant groups as measured by flow cytometry.** **(A, D)** Cross-sectional profiling of the proportions of TIM3^+^LAG3^+^ CD8^+^ T cells, and TIM3^+^LAG3^+^ CD8^-^ T cells, respectively, between the VL-HIV, the *Leishmania*-seropositive HIV, and the HIV-only groups, using a Benjamini-Hochberg corrected pairwise Mann-Whitney U test to test for statistical differences. In the latter group, the plus sign (+) indicates those *Leishmania*-seropositive individuals with a history of VL. **(B, E)** Comparison between chronic and non-chronic VL-HIV patients at the active disease development (D0) and End-of-Treatment (EOT) timepoints using a BH-corrected Mann-Whitney U to test for statistical differences **(C, F)** Longitudinal characterisation of the proportions of the same cellular subsets, for non-chronic or chronic VL-HIV patients only, using linear mixed-effects models (see Methods). Cross-sectional analysis total N = 62. Longitudinal analysis N = 24.


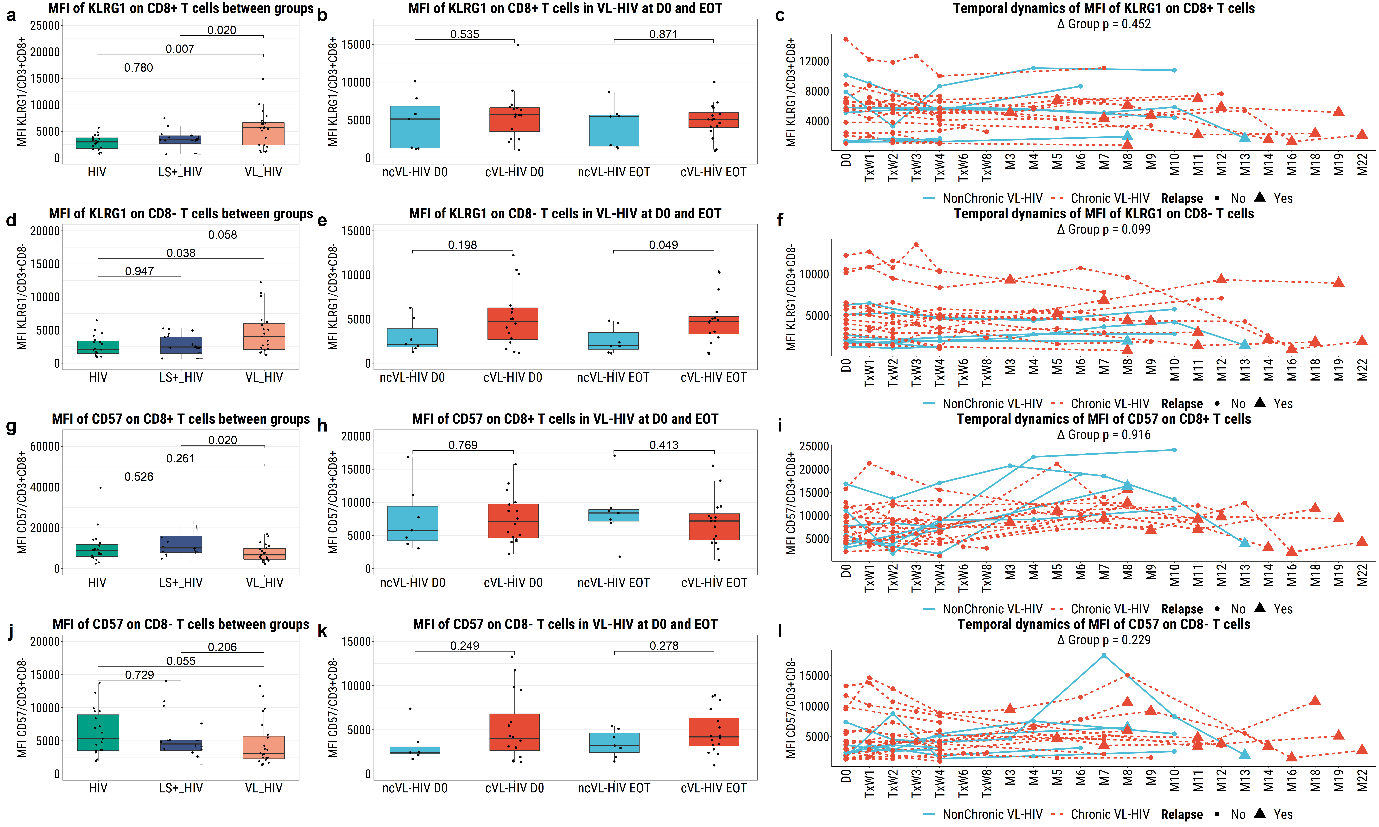


**Supplementary Figure 8 | Immunosenescence in the CD8^+^ T cell (CD3^+^CD8^+^) and CD8^-^ T cell (CD3^+^CD8^-^) fractions of PBMCs isolated from the different patient groups as measured by flow cytometry. (A, D, G, J)** Cross-sectional profiling of the MFIs of KLRG1 on CD8^+^ T cells, KLRG1 on CD8^-^ T cells, CD57 on CD8^+^ T cells, and CD57 on CD8^-^ T cells, respectively, using a Benjamini-Hochberg corrected pairwise Mann-Whitney U test to test for statistical differences. In the LS+-HIV group, the plus sign (+) indicates those *Leishmania*-seropositive individuals with a history of VL. **(B, E, H, K)** Longitudinal characterisation of the MFIs of the same cellular subsets, for primary and chronic VL-HIV patients only, using linear mixed-effects models (see Methods). **(C, F, I, L)** Characterisation of the MFIs of the same cellular subsets, in function of the annualised relapse rate, using linear mixed-effects models. Cross-sectional analysis total N = 62. Longitudinal analysis N = 24.


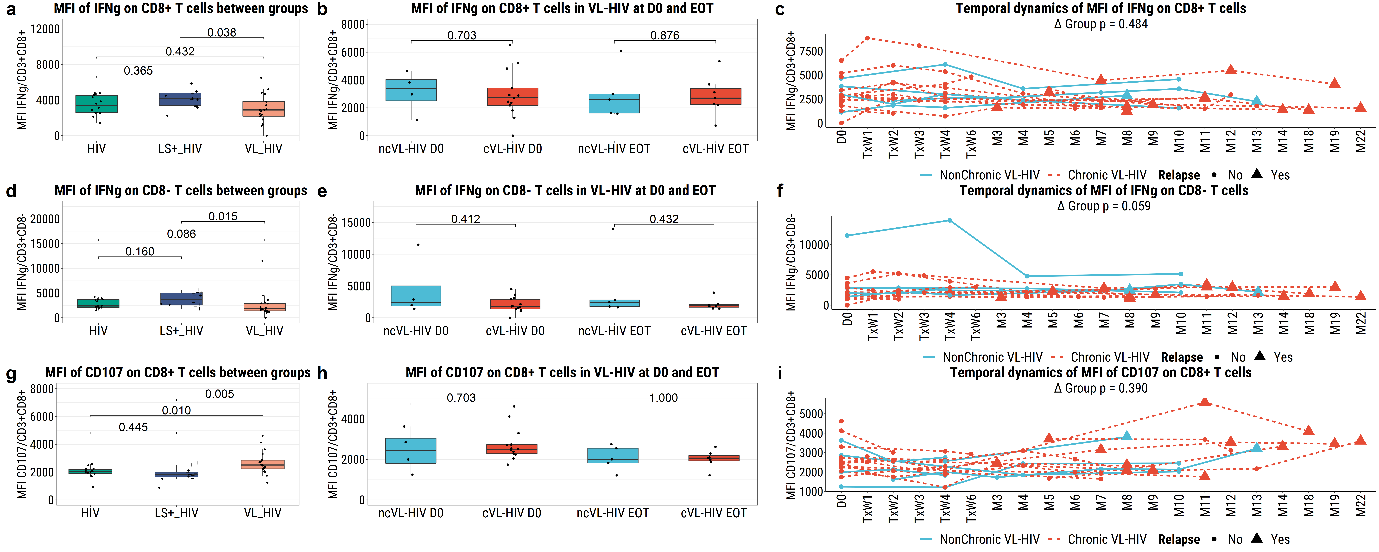


**Supplementary Figure 09 | Functionality markers on the CD8^+^ T cell (CD3^+^CD8^+^) and CD8^-^ T cell (CD3^+^CD8^-^) fractions of PBMCs isolated from the different patient groups as measured by flow cytometry. (A, D, G)** Cross-sectional profiling of the MFIs of IFNγ on CD8^+^ T cells and CD8^-^ T cells, and CD107a on CD8^+^ T cells, respectively, using a Benjamini-Hochberg corrected pairwise Mann-Whitney U test to test for statistical differences. In the LS+-HIV group, the plus sign (+) indicates those *Leishmania*-seropositive individuals with a history of VL. **(B, E, H)** Longitudinal characterisation of the MFIs of the same cellular subsets, for primary and chronic VL-HIV patients only, using linear mixed-effects models (see Methods). **(C, F, I)** Characterisation of the MFIs of the same cellular subsets, in function of the annualised relapse rate, using linear mixed-effects models. Cross-sectional analysis total N = 62. Longitudinal analysis N = 24.


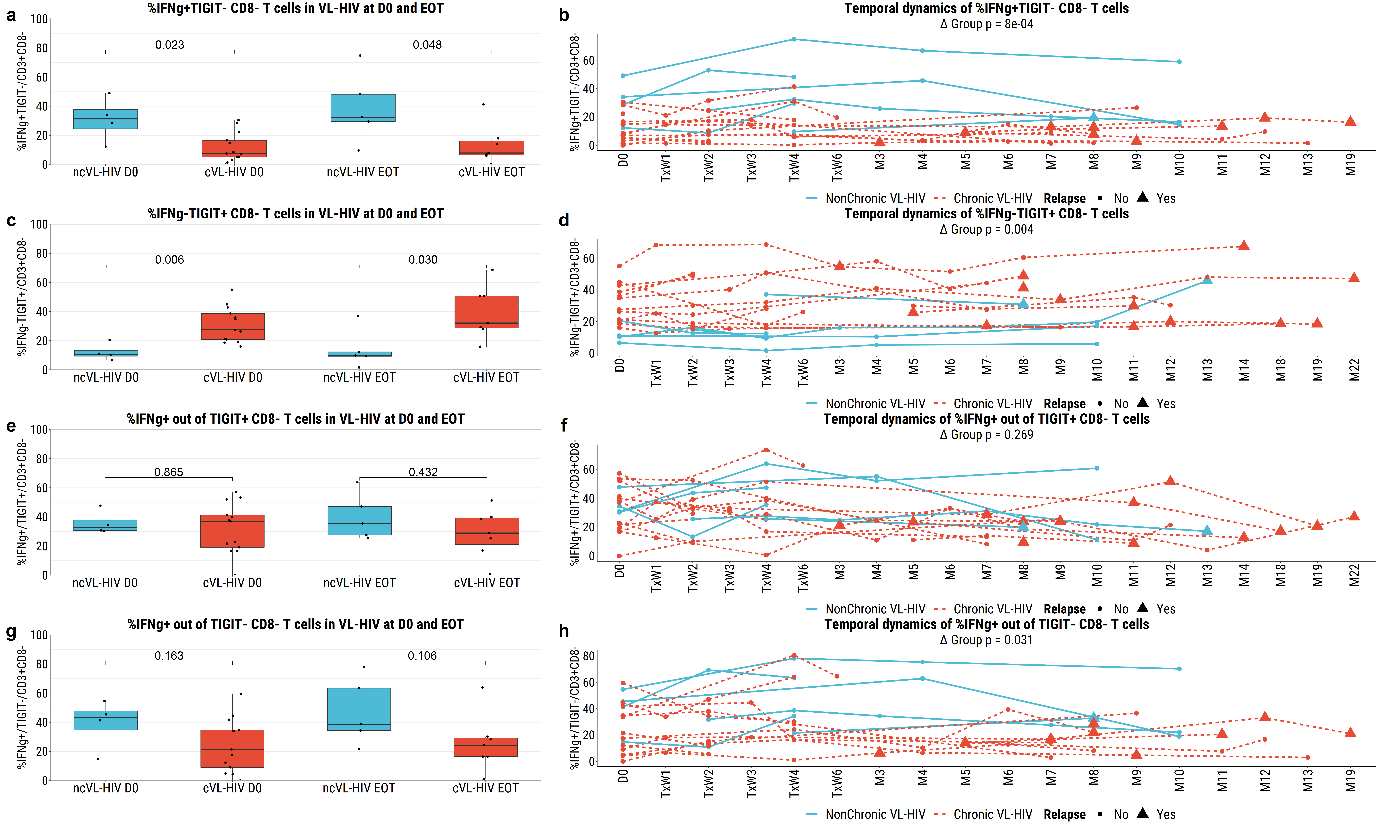


**Supplementary Figure 10 | Interferon-γ positivity in the TIGIT^+^ and TIGIT^-^ CD8^-^ (CD3^+^CD8^-^) T cell fractions of PBMCs isolated from the different participant groups as measured by flow cytometry.** **(A, C, E, G)** Comparison of the proportions of IFN-γ^+^TIGIT^-^ cells out of all CD8^-^ T cells, IFN-γ^-^TIGIT^+^ cells out of all CD8^-^ T cells, IFN-γ^+^ cells out of TIGIT^+^CD8^-^ T cells, and IFN-γ^+^ cells out of TIGIT^-^CD8^-^ T cells, respectively, between the chronic (n=13 at D0, n=7 at EOT) and non-chronic (n=4 at D0, n=5 at EOT) VL-HIV patients at the active disease development (D0) and End-of-Treatment (EOT) timepoints using a BH-corrected Mann-Whitney U to test for statistical differences. **(B, D, F, H)** Longitudinal characterisation of the proportions of the same cellular subsets, for non-chronic or chronic VL-HIV patients, using linear mixed-effects models (see Methods).’


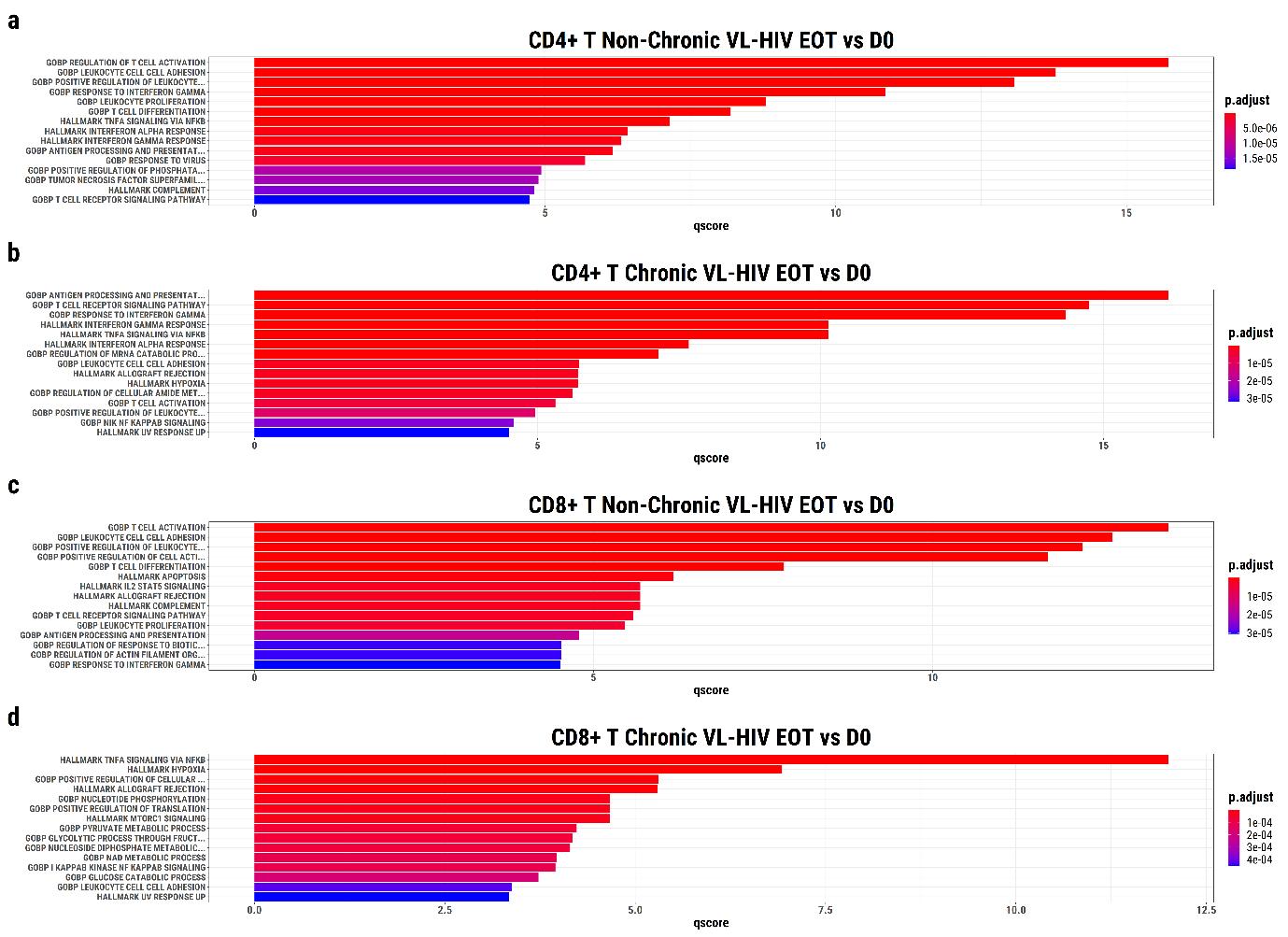


**Supplementary Figure 11 | Enrichment scores of pathway overrepresentation analysis performed on the differentially expressed genes between** **(A)** CD4^+^ T cells of non-chronic VL-HIV at End-of-Treatment (EOT) versus active disease (D0), **(B)** CD4^+^ T cells of chronic VL-HIV at EOT versus D0, **(C)** CD8^+^ T cells of non-chronic VL-HIV patients at EOT versus D0, (**D)** CD8^+^ T cells of chronic VL-HIV patients at EOT versus D0. N = 2 each group and timepoint.


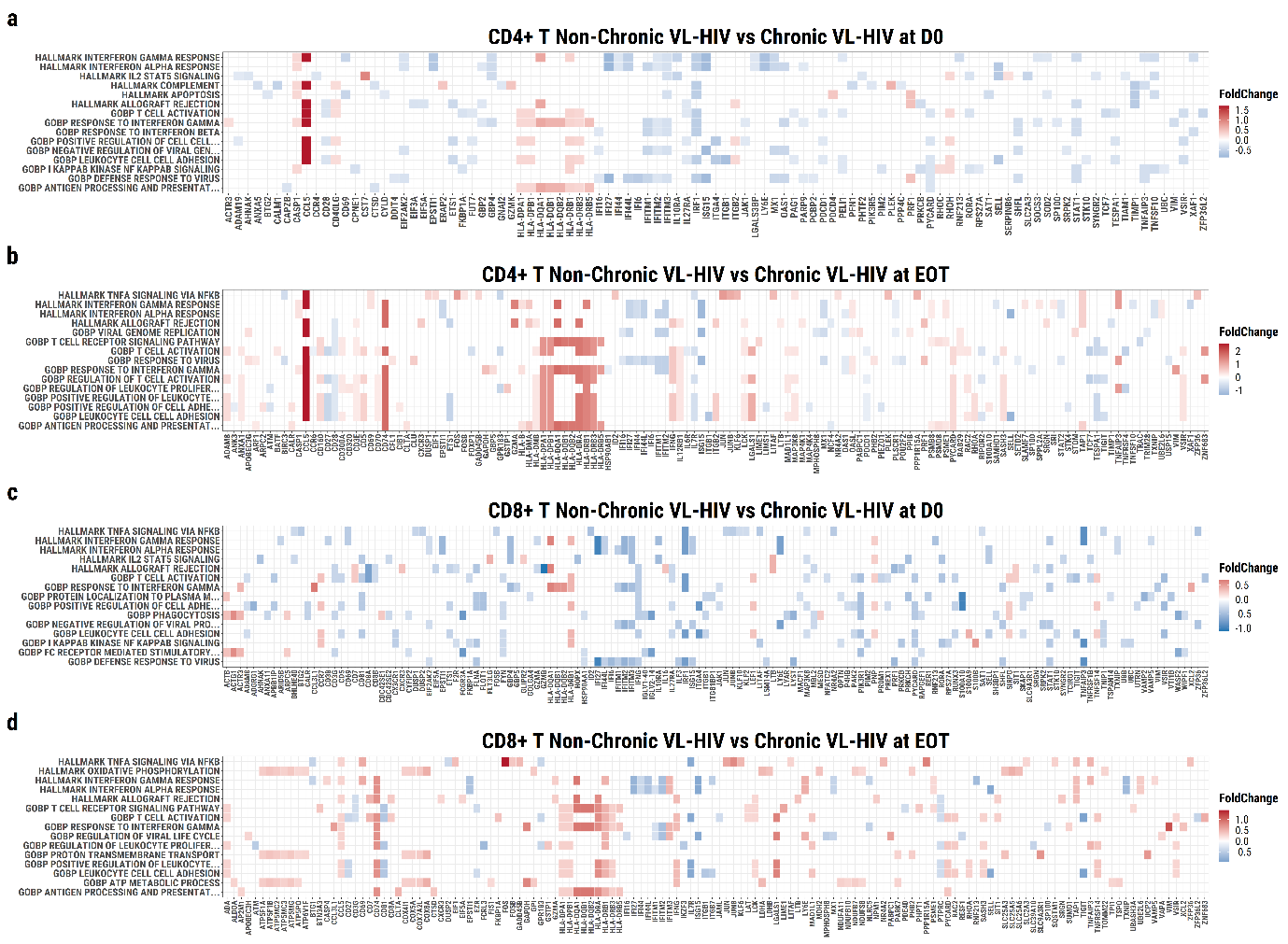


**Supplementary Figure 12 | Pathway overrepresentation visualised as a heatmap performed on the differentially expressed genes between** **(A)** CD4^+^ T cells of non-chronic VL-HIV patients and chronic VL-HIV patients at D0, **(B)** CD4^+^ T cells of non-chronic VL-HIV patients and chronic VL-HIV patients at EOT, **(C)** CD8^+^ T cells of non-chronic VL-HIV patients and chronic VL-HIV patients at D0, (**D)** CD8^+^ T cells of non-chronic VL-HIV patients and chronic VL-HIV patients at EOT. N = 2 each group and timepoint.

**Supplementary Table 1 | Participant socio-demographic, clinical, and biochemical characteristics**, **for the participant subset included in the single-cell analyses**, at various timepoints, including at time of recruitment for HIV and AL-HIV (LS+-HIV with no VL history) patients or active disease development for VL-HIV patients (D0), at End-of-Treatment (EOT; VL-HIV only) and at six months post-treatment (Post M6; VL-HIV only). Participant characteristics for the healthy endemic controls (HEC) are not reported.

|  | Total (n=8) | HIV (n=2) | AL-HIV (n=2) | VL-HIV (n=4) | Non-chronic VL-HIV (n=2) | Chronic VL-HIV (n=2) |
| --- | --- | --- | --- | --- | --- | --- |
| Socio-demographic characteristics |  |  |  |  |  |  |
| Age in years, median (IQR) | 42.5 (36.5-45.5) | 39 (37-41) | 31 (28-34) | 46 (44-52.5) | 58 (52.5-63.5) | 43.5 (43-44) |
| Male, n (%) | 8 (100) | 2 (100) | 2 (100) | 4 (100) | 2 (100) | 2 (100) |
| BMI in kg/m2, median (IQR) | 17.6 (15.8-19) | 17.5 (17.1-18) | 16.8 (15.7-17.8) | 17.6 (15.8-19.3) | 17.6 (16.7-18.4) | 17.6 (16.7-18.5) |
| Literacy, n (%) | 6 (75) | 1 (50) | 2 (100) | 3 (75) | 2 (100) | 1 (50) |
| Daily labourer | 5 (62.5) | 0 (0) | 2 (100) | 3 (75) | 1 (50) | 2 (100) |
| Farmer | 1 (12.5) | 0 (0) | 0 (0) | 1 (25) | 1 (50) | 0 (0) |
| Other | 2 (25) | 2 (100) | 0 (0) | 0 (0) | 0 (0) | 0 (0) |
| Clinical history and characteristics | | | | | | |
| VL history, n (%) | 2 (25) | 0 (0) | 0 (0) | 2 (50) | 0 (0) | 2 (100) |
| Past VL episodes, median (IQR) | 0 (0-2.25) | 0 (0-0) | 0 (0-0) | 4.5 (0-9.5) | 0 (0-0) | 10 (9.5-10.5) |
| Months since previous VL episode, median (IQR) | 3.5 (3-4) | NA | NA | 3.5 (3-4) | NA | 3.5 (3-4) |
| Microscopically confirmed, n (%) | 2 (25) | 0 (0) | 0 (0) | 2 (50) | 0 (0) | 2 (100) |
| Parasite grading, n (%) | | | | | | |
| +4 to +6 | 2 (25) | 0 (0) | 0 (0) | 2 (50) | 0 (0) | 2 (100) |
| VL treatment regimen, n (%) | | | | | | |
| AmBisome + Miltefosine | 4 (50) | 0 (0) | 0 (0) | 4 (100) | 2 (100) | 2 (100) |
| On ART, n (%) | 8 (100) | 2 (100) | 2 (100) | 4 (100) | 2 (100) | 2 (100) |
| Concomitant diseases, n (%) | 0 (0) | 0 (0) | 0 (0) | 0 (0) | 0 (0) | 0 (0) |
| Laboratory markers at D0 |  |  |  |  |  |  |
| CD4 count, median (IQR); (cells/μl) | 100 (73-238) | 416 (296-536) | 249 (164-334)) | 76 (55-97) | 54 (52-55) | 100 (97-102) |
| Lymphocytes, median (IQR); (x10^3^/µl) | 0.7 (0.26-1.08) | 1.31 (1.19-1.44) | 0.93 (0.83-1.04) | 0.24 (0.19-0.38) | 0.17 (0.15-0.19) | 0.48 (0.38-0.57) |
| Platelets, median (IQR); (x10^3^/µl) | 140 (107-145) | 142 (138-145) | 136 (91-181) | 136 (100-144) | 136 (131-140) | 82 (52-113) |
| Hemoglobin, median (IQR); (g/dL) | 10 (7.9-11.3) | 13.6 (12.2-14.9) | 9.6 (9-10.1) | 7.8 (6.1-10.1) | 5.9 (5.7-6.1) | 10.9 (10.1-11.7) |
| Laboratory markers at EOT |  |  |  |  |  |  |
| Lymphocytes, median (IQR); (x10^3^/µl) | NA | NA | NA | 0.57 (0.28-0.89) | 0.49 (0.32-0.65) | 0.73 (0.52-0.93) |
| Platelets, median (IQR); (x10^3^/µl) | NA | NA | NA | 184 (140-242) | 282 (242-322) | 111 (82-140) |
| Hemoglobin, median (IQR); (g/dL) | NA | NA | NA | 10 (8.5-11.3) | 8 (7.5-8.5) | 11.7 (11.3-12) |
| Laboratory markers at Post M6 |  |  |  |  |  |  |
| CD4 count, median (IQR); (cells/μl) | NA | NA | NA | 273 (210-448) | 448 (360-536) | 146 (146-146)* |
| Lymphocytes, median (IQR); (x10^3^/µl) | NA | NA | NA | 0.78 (0.74-1.04) | 1.04 (0.91-1.16) | 0.7 (0.7-0.7)* |
| Platelets, median (IQR); (x10^3^/µl) | NA | NA | NA | 158 (124-231) | 272 (231-314) | 122 (120-124) |
| Hemoglobin, median (IQR); (g/dL) | NA | NA | NA | 10.4 (9.6-11.3) | 10.2 (9.8-10.7) | 10.6 (10.2-11.1) |

NA = Not Applicable. * = missing value for one out of two patients.

**Supplementary Table 2 | Participant socio-demographic, clinical, and biochemical characteristics,** at study inclusion for HIV and LS+-HIV participants, or at active disease development for individuals with VL-HIV (D0).

|  | Total (n=63) | HIV (n=19) | LS+-HIV (n=20) | VL-HIV (n=24) | *P*-value |
| --- | --- | --- | --- | --- | --- |
| Socio-demographic characteristics | | | | | |
| Age in years, median (IQR) | 39 (34-44.5) | 39 (34-43) | 39 (35-46) | 39.5 (31-42) | 0.827 |
| Male, n (%) | 52 (82.5) | 8 (42.1) | 20 (100) | 24 (100) | <0.001 |
| BMI in kg/m2, median (IQR) | 18 (16-19.7) | 20 (17.6-21) | 17.9 (16.8-18.8) | 16.6 (15.4-18.6) | 0.004 |
| Literacy, n (%) | 36 (57.1) | 9 (47.4) | 10 (50) | 17 (70.8) | 0.251 |
| Occupation, n (%) | | | | | 0.014 |
| Daily labourer | 30 (47.6) | 5 (26.3) | 10 (50) | 15 (62.5) |  |
| Farmer | 18 (28.6) | 4 (21.1) | 8 (40) | 6 (25) |  |
| Other | 15 (23.8) | 10 (52.6) | 2 (10) | 3 (12.5) |  |
| Clinical characteristics and disease history | | | | | |
| VL history, n (%) | 32 (50.8) | 0 (0) | 13 (65) | 19 (79.2) | NA |
| Past VL episodes, median (IQR) | 1 (0-2) | 0 (0-0) | 1 (0-2) | 2 (1-6) | NA |
| Months since previous VL episode, median (IQR) | 9.5 (4-80) | NA | 73 (9-135) | 6 (4-15.5) | NA |
| On ART, n (%) | 62 (98.4) | 19 (100) | 20 (100) | 23 (95.8) | 1 |
| Concomitant diseases, n (%) | 7 (11.1) | 0 (0) | 3 (15) | 4 (16.7) | 0.177 |
| Laboratory markers | | | | | |
| rK39 RDT positivity, n (%) | 43 (68.3) | 0 (0) | 20 (100) | 23 (95.8) | NA |
| rK39 ELISA positivity, n (%) | 39 (61.9) | 0 (0) | 18 (90) | 21 (87.5) | NA |
| KAtex positivity, n (%) | 22 (34.9) | 1 (5.3) | 2 (10) | 19 (79.2) | NA |
| DAT positivity, n (%) | 40 (63.5) | 0 (0) | 18 (90) | 22 (91.7) | NA |
| *Leishmania* PCR positivity, n (%) | 26 (41.3) | 0 (0) | 2 (10) | 24 (100) | NA |
| CD4 count, median (IQR); (cells/μl) | 259 (99.5-421) | 425 (262-750) | 342 (221-420) | 58 (37-126) | <0.001 |
| Lymphocytes, median (IQR); (x10^3^/µl) | 1.02 (0.67-1.53) | 1.61 (1.06-1.99) | 1.11 (0.87-1.31) | 0.51 (0.3-0.79) | <0.001 |
| Platelets, median (IQR); (x10^3^/µl) | 165 (122.2-245.8) | 245 (199-300) | 204 (156-278) | 114 (85-138) | <0.001 |
| Hemoglobin, median (IQR); (g/dL) | 12.05 (9.73-13.78) | 13.8 (13.1-14.3) | 12.3 (11.8-14.3) | 9.4 (8.6-10.1) | <0.001 |

NA = Not Applicable.
