# Supplementary material for: Persistent T cell unresponsiveness associated with chronic visceral leishmaniasis in HIV-coinfected patients
Source: Commun Biol. 2024 May 3;7:524. doi: 10.1038/s42003-024-06225-2 (PMC11068874; doi:10.1038/s42003-024-06225-2)
Supplement: Supplementary file 3 — Description of Additional Supplementary Files [file 42003_2024_6225_MOESM3_ESM.pdf]

Description of Additional Supplementary Files

File name: Supplementary Data 1

Description: Flow cytometry data - The source data behind the flow cytometry graphs in the manuscript.
