# Supplementary material for: Persistent T cell unresponsiveness associated with chronic visceral leishmaniasis in HIV-coinfected patients
Source: Commun Biol. 2024 May 3;7:524. doi: 10.1038/s42003-024-06225-2 (PMC11068874; doi:10.1038/s42003-024-06225-2)
Supplement: Supplementary file 5 — Reporting Summary [file 42003_2024_6225_MOESM5_ESM.pdf]

nature portfolio

Corresponding author(s): Wim Adriaensen

Last updated by author(s): Mar 25, 2024

Reporting Summary

Nature Portfolio wishes to improve the reproducibility of the work that we publish. This form provides structure for consistency and transparency in reporting. For further information on Nature Portfolio policies, see our Editorial Policies and the Editorial Policy Checklist.

Statistics

For all statistical analyses, confirm that the following items are present in the figure legend, table legend, main text, or Methods section.

n/a	Confirmed	
☐	☒	The exact sample size (*n*) for each experimental group/condition, given as a discrete number and unit of measurement
☐	☒	A statement on whether measurements were taken from distinct samples or whether the same sample was measured repeatedly
☐	☒	The statistical test(s) used AND whether they are one- or two-sided<br>*Only common tests should be described solely by name; describe more complex techniques in the Methods section.*
☒	☐	A description of all covariates tested
☐	☒	A description of any assumptions or corrections, such as tests of normality and adjustment for multiple comparisons
☐	☒	A full description of the statistical parameters including central tendency (e.g. means) or other basic estimates (e.g. regression coefficient) AND variation (e.g. standard deviation) or associated estimates of uncertainty (e.g. confidence intervals)
☒	☐	For null hypothesis testing, the test statistic (e.g. *F*, *t*, *r*) with confidence intervals, effect sizes, degrees of freedom and *P* value noted<br>*Give P values as exact values whenever suitable.*
☒	☐	For Bayesian analysis, information on the choice of priors and Markov chain Monte Carlo settings
☒	☐	For hierarchical and complex designs, identification of the appropriate level for tests and full reporting of outcomes
☒	☐	Estimates of effect sizes (e.g. Cohen's *d*, Pearson's *r*), indicating how they were calculated

Our web collection on statistics for biologists contains articles on many of the points above.

Software and code

Policy information about availability of computer code

Data collection	Electronic Data capture was done with the web-based MACRO EDC system for clinical trials.
Data analysis	Initial flow cytometry data analysis was carried out in FlowJo v8.5. Subsequently, all flow cytometry data was further analysed in R version 4.1.1. Packages used include: here 1.0.1, dplyr 1.0.8, stringr 1.4.0, tidyr 1.2.0, forcats 0.5.1, pheatmap 1.0.12, ggsci 2.9, ggpubr 0.4.0, rstatix 0.7.0, gridExtra 2.3, patchwork 1.1.1, gplots 3.1.1, ggrepel 0.9.1, ggraph 2.0.5, hrbrthemes 0.8.0, extrafont 0.17, Cairo 1.5-12.2, lmerTest 3.1-3. Raw single-cell sequencing data was first processed with CellRanger v6.0.0, and then analysed in R v4.2.1. For single-cell analyses, additional R packages used for analyses include: geomtextpath 0.1.1, org.Hs.eg.db 3.14.0, clusterprofiler 4.2.0, enrichplot 1.14.1, scRepertoire 1.7.2, reticulate 1.2.4, Seurat 4.1.0, scGate 1.0.0, msigdbr 7.4.1 and AnnotationDbi 1.56.2. Custom code for data analysis has been deposited on a dedicated GitHub repository at https://github.com/BioinformaNicks/PreLeisH_Immuno

For manuscripts utilizing custom algorithms or software that are central to the research but not yet described in published literature, software must be made available to editors and reviewers. We strongly encourage code deposition in a community repository (e.g. GitHub). See the Nature Portfolio guidelines for submitting code & software for further information.

Data

Policy information about availability of data

All manuscripts must include a data availability statement. This statement should provide the following information, where applicable:

- Accession codes, unique identifiers, or web links for publicly available datasets
- A description of any restrictions on data availability
- For clinical datasets or third party data, please ensure that the statement adheres to our policy

Processed single-cell RNA and T cell receptor sequencing data has been deposited on Zenodo at https://doi.org/10.5281/zenodo.10102745. Raw sequencing data is only available on request due to privacy concerns. Flow cytometry values used in the analyses have been provided in the Supplementary Data 1 file. Raw flow cytometry data is also available on request. Patient characteristics data will remain restricted due to privacy and ethics concerns, however, summary statistics have been provided in the manuscript.

Research involving human participants, their data, or biological material

Policy information about studies with human participants or human data. See also policy information about sex, gender (identity/presentation), and sexual orientation and race, ethnicity and racism.

Reporting on sex and gender	As visceral leishmaniasis disproportionately affects the male sex, there are no females included in the Leishmania co-infected groups. In the HIV group, 11 females are included. No stratification or analysis was done by sex as only a limited number of female participants was included, and the main focus was on analyses of Leishmania co-infected individuals of which all were male. Gender data was not gathered.
Reporting on race, ethnicity, or other socially relevant groupings	We did not do any grouping by race, ethnicity, or by other socially relevant groupings. All participants were from North-West Ethiopia.
Population characteristics	Covariates reported include: Age, Sex, Literacy, BMI, Occupation, Visceral leishmaniasis disease history, Antiretroviral therapy usage, Visceral leishmaniasis treatment regimen data, Concomitant diseases, rk39 antigen test results (ELISA and Rapid Diagnostic Test), Direct Agglutination Test results, Leishmania PCR results, CD4 T cell counts, Lymphocyte count, Platelet count and Hemoglobin levels.
Recruitment	All eligible patients attending ART services at one of the study sites were invited consecutively to participate in the PreLeisH study (NCT03013673). Due to a high prevalence of VL in male participants, we aimed for a stratified recruitment status by sex (80% males, 20% females). All patients testing positive for rK39 RDT at baseline and every fifth patient were included in the immune subgroup, that included PBMC isolation and biobanking an all subsequent visits.
Ethics oversight	The study protocol was approved by the Ethiopian National Research Ethics Review Committee, the University of Gondar Institutional Review Board, the Institute of Tropical Medicine Antwerp Institutional Review Board, the Médecins Sans Frontiers Ethics Review Board and the Antwerp University Hospital Ethics Committee. All patients provided written informed consent and the study was carried out in accordance with international guidelines (Helsinki declaration, Good Clinical Practices and local regulations).

Note that full information on the approval of the study protocol must also be provided in the manuscript.

Field-specific reporting

Please select the one below that is the best fit for your research. If you are not sure, read the appropriate sections before making your selection.

☒ Life sciences ☐ Behavioural & social sciences ☐ Ecological, evolutionary & environmental sciences

For a reference copy of the document with all sections, see nature.com/documents/nr-reporting-summary-flat.pdf

Life sciences study design

All studies must disclose on these points even when the disclosure is negative.

Sample size	This study was embedded within a large cohort study to predict VL development (NCT03013673). A convenience sampling was performed based was on available samples and metadata at the time.
Data exclusions	Samples from patients were excluded from the flow cytometry analysis if they were below 25% viability.
Replication	No measures were taken to replicate the experimental findings due to a lack of additional samples. However, repeated measures of the same individuals were taken and were coherent with eachother.
Randomization	No randomizaton was performed as no intervention was performed.
Blinding	No study blinding was performed, but samples were processed in the lab independent from patient metadata.

Reporting for specific materials, systems and methods

We require information from authors about some types of materials, experimental systems and methods used in many studies. Here, indicate whether each material, system or method listed is relevant to your study. If you are not sure if a list item applies to your research, read the appropriate section before selecting a response.

Materials & experimental systems

n/a	Involved in the study
☒	☐ Antibodies
☒	☐ Eukaryotic cell lines
☒	☐ Palaeontology and archaeology
☒	☐ Animals and other organisms
☐	☒ Clinical data
☒	☐ Dual use research of concern
☒	☐ Plants

Methods

n/a	Involved in the study
☒	☐ ChIP-seq
☐	☒ Flow cytometry
☒	☐ MRI-based neuroimaging

Clinical data

Policy information about clinical studies

All manuscripts should comply with the ICMJE guidelines for publication of clinical research and a completed CONSORT checklist must be included with all submissions.

Clinical trial registration: NCT03013673

Study protocol: The study protocol can be found at clinicaltrials.gov

Data collection: Conform to international regulations and GCP, data was continuously collected throughout the study period and entered in the MACRO database, with regular monitoring, data review and source data verification.

Outcomes: We longitudinally characterised the compositional and functional changes in peripheral immune cell subsets, as blood represents an easily accessible compartment for clinical care, in non-chronic and chronic VL-HIV patients over a median of 19.5 months and compared it to long-term cured or asymptomatic Leishmania-infected individuals and a HIV control group. In this substudy, we included the first 24 participants that developed active VL during the study period between 2017 and 2019 (excluding those lacking blood sampling) (Figure 1A). These 24 patients were stratified in non-chronic patients (ncVL-HIV; n=7), that included five individuals with no VL history and two with one prior VL episode more than 10 years (with a median of 185 (IQR 164-206) months) before enrollment in our study, and chronic patients (cVL-HIV; n=17) with one or more recent VL episodes all within 3 years (with a median of 5 (IQR 4-10) months) prior to study recruitment. As control groups, we included HIV-positive participants with no VL history and no asymptomatic Leishmania infection over the complete study period (HIV; n=19; based on consecutive and confirmatory sampling); and a mixture of asymptomatic Leishmania-infected and long-term VL cured HIV-positive participants that tested positive for at least 2 or more Leishmania markers (rK39 RDT, rK39 ELISA, DAT, KATEX, PCR) at consecutive timepoints during the study period (Leishmania-seropositive-HIV, LS+-HIV; n=20; consecutive sampling).

Plants

Seed stocks: NA

Novel plant genotypes: NA

Authentication: NA

Flow Cytometry

Plots

Confirm that:

- [ ] The axis labels state the marker and fluorochrome used (e.g. CD4-FITC).
- [x] The axis scales are clearly visible. Include numbers along axes only for bottom left plot of group (a 'group' is an analysis of identical markers).
- [ ] All plots are contour plots with outliers or pseudocolor plots.
- [x] A numerical value for number of cells or percentage (with statistics) is provided.

Methodology

Sample preparation	Thawed PBMCs were washed and subjected to two separate antibody panels (Miltenyi Biotec, USA), according to cell availability. The CD8+ T-cell exhaustion-targeted panel consisted of anti-CD3-VioGreen, anti-CD8-VioBlue, anti-CD57-APC-Vio770, anti-LAG-3-APC, anti-KLRG1-PE-Vio770, anti-PD-1-PE, anti-TIM-3-VioBright-FITC, and 7AAD for viability. The CD8+ T-cell functionality-targeted panel consisted of anti-CD3-VioGreen, anti-CD8-VioBlue, anti-IFNγ-Vio667, anti-CD95-PE-Vio770, anti-TIGIT-PE, anti-CD107α-FITC, and FVS780 for viability. For the CD8+ T cell functionality-targeted panel, cells were thawed, washed and pre-stimulated with 1 µl of Leukocyte Activation Cocktail with GolgiPlug (LAC, BD Biosciences, Belgium) per 300.000 cells, for 4 hours in a humidified incubator at 5% CO2 and 37°C. In addition to this, anti-CD107α antibody was immediately added to the cells with LAC (with GolgiPlug), and after 1 hour, GolgiStop was added for the remaining 3 hours of incubation. Afterwards, cells were fixed and permeabilised before intracellular co-staining using the FOXP3/Transcription Factor Staining Buffer Set (eBioscience, USA) according to the manufacturer's instructions. After fixation/permeabilisation, cells were washed and immediately acquired on a FACSVerse flow cytometer (BD Biosciences, San Jose, CA), including prior FMO controls and single stainings for each parameter. The gating strategy is outlined in Supplementary Figures S1-2
Instrument	FACSVerse flow cytometer (BD Biosciences, San Jose, CA)
Software	Initial analysis was performed using FlowJo v8.5 and the cellular subsets were expressed as proportions. Subsequent analysis was performed in R v4.1.1
Cell population abundance	*Describe the abundance of the relevant cell populations within post-sort fractions, providing details on the purity of the samples and how it was determined.*
Gating strategy	Patient PBMCs were isolated and stained for flow cytometry. For the first panel, after exclusion of doublets and non-lymphocytes, single lymphocytes were gated for live cells. Next, CD8+ and CD8- T cells were gated out by plotting CD3-VioGreen versus CD8-VioBlue. Out of these subsets, those positive for a range of exhaustion and senescence markers (CD57-APC-Vio770, LAG3-APC, KLRG1-PE-Vio770, PD-1-PE, TIM3-VioBright-FITC) were gated out. In addition, those double-positive for PD-1-PE, TIM3-VioBright-FITC), and LAG3-APC were gated out.<br>For the second panel, after exclusion of doublets and non-lymphocytes, single lymphocytes were gated for live cells. Next, CD8+ and CD8- T cells were gated out by plotting CD3-VioGreen versus CD8-VioBlue. Out of these subsets, those positive for TIGIT-PE, IFNγ-Vio667, and CD107a-FITC were gated out. In addition, double-positive, double-negative or positive-negative for TIGIT-PE and IFNγ-Vio667 was gated out.

- [x] Tick this box to confirm that a figure exemplifying the gating strategy is provided in the Supplementary Information.
